# The Relationship between Motor Coordination and Imitation: An *f*NIRS Study

**DOI:** 10.3390/brainsci11081052

**Published:** 2021-08-09

**Authors:** Wenrui Zhao, Minqiang Hui, Xiaoyou Zhang, Lin Li

**Affiliations:** 1 Key Laboratory of Adolescent Health Assessment and Exercise Intervention of the Ministry of Education, East China Normal University, Shanghai 200241, China; zhaowenrui108@163.com (W.Z.); m13213595838@163.com (M.H.); xiaoyouzhang@vip.163.com (X.Z.); 2 College of Physical Education and Health, East China Normal University, Shanghai 200241, China; 3 Qiushi College, Taiyuan University of Technology, Jinzhong 030600, China

**Keywords:** motor coordination, action imitation, functional near infrared spectroscopy imaging (*f*NIRS), mirror neuron system

## Abstract

Although motor coordination and imitation are important factors affecting motor skill learning, few studies have examined the relationship between them in healthy adults. In order to address this in the present study, we used *f*NIRS to analyze the relationship between motor coordination and imitation in college students. Our results showed that: (1) motor coordination in female students was positively correlated with the average time taken to perform an imitation; (2) the mean imitation time was negatively correlated with the activation level of the supplementary motor cortex, primary somatosensory cortex, and angular gyrus of the mirror neuron system; (3) motor coordination in female students moderated mirror neuron system (MNS) activation and imitation. For women with low rather than high motor coordination, higher MNS activation was associated with a stronger imitation ability. These results demonstrate that motor coordination in female students is closely related to action imitation, and that it moderates the activation of the MNS, as measured via *f*NIRS.

## 1. Introduction

Action imitation emerges in infancy [1] and is present in many aspects of life, especially in the process of motor skill learning. Imitation is the repetition of observed physical movements [2]. In motor skill learning, a learner first observes the movement of a demonstrator, and is then gradually able to produce movements that are consistent with the demonstrator’s movements through repeated exercise [3]. When learning new motor skills, an individual’s motor coordination plays an important role. Motor coordination refers to the timing of interactions between different physical systems, body parts, and organs in order to achieve a unified action. Motor coordination is an important basis for the formation of motor skills, and requires comprehensive interactions between different neural systems [4]. Human motor coordination is involved in generating reflexive responses, spatial orientation, ontological perception, rhythm perception and production, balance, action cognition, and so on [4].

Despite the close connection between imitation and motor coordination, few studies have examined this relationship. Further, these studies have been limited to infants and children with developmental coordination disorder (DCD). Researchers examining the development of movement in infants have found that those with better movement coordination are more quickly able to imitate and complete grasping movements [5,6]. Compared with healthy children, children with DCD may have difficultly imitating simple and complex gestures [7]. Thus, there is a close relationship between motor coordination and imitation in infants and children. However, whether this connection is also present in healthy adults has not yet been established.

At the end of the 20th century, researchers began to examine the neural mechanisms of imitation using neuroimaging technology (magnetic resonance imaging, electroencephalogram, etc.). When people observe, imagine, perform, and imitate actions, the mirror neuron system (MNS), comprising multimodal neurons in the frontal and parietal lobes, is triggered [8,9]. The MNS was first identified in the premotor cortex (F5) and the rostral side (PFG) of the inferior parietal lobule (IPL) in non-human primates [10]. Since then, researchers have explored the MNS in humans. MNS activity responsible for human imitation has been found to include the dorsal premotor cortex (PMD), ventral premotor cortex (PMV), intraparietal sulcus (AIPS), and superior marginal gyrus (SMG), which together constitute the core circuit of visual–motor transmission. In addition, sensorimotor regions, including the inferior frontal gyrus (IFG), supplementary motor area (SMA), and angular gyrus (ANG), also play a role [11]. Reynolds et al. examined brain activation when both children with DCD and healthy children observed, performed, and imitated finger tasks, and found differences in the activation of brain regions related to mirror neurons between children with DCD and healthy children during imitation. Thus, MNS dysfunction may be the basis of imitation and movement disorders in children [12]. Another study explored the activation of brain regions in children with DCD and healthy children during imitation, and found that posterior inferior frontal gyrus activation was lower in children with DCD [7]. Current studies have shown that, compared with healthy children, those with DCD not only exhibit defects in imitation, but also show altered MNS activity in regions responsible for imitation.

Most previous studies examining the neural basis of imitation have used magnetic resonance imaging (MRI) [8,9]. However, subjects must lie down during data collection, which limits the range of actions that can be performed in an imitation task. Functional near-infrared spectroscopy (*f*NIRS) has the advantages of a high temporal resolution and spatial resolution, and is not as sensitive as MRI to the motor actions of subjects during scanning [13,14,15]. Thus, it is a more convenient choice for the collection of neural data from healthy adults during an action simulation task.

In this study, we used *f*NIRS to examine changes in oxyhemoglobin (HbO) in the frontal and parietal cortex during imitation. Our goal was to elucidate the relationship between motor coordination and imitation in healthy adults in terms of behavior and neural activation. We hypothesized that there would be a correlation between motor coordination and imitation in healthy adults, that the MNS would be the neural basis of imitation-related behaviors, and that different types of motor coordination would have different effects on MNS activation.

## 2. Materials and Methods

### 2.1. Subjects

We recruited 53 college students (24 men and 29 women). All subjects were right-handed, had no diseases affecting brain function or structure, had no mental health conditions (no anxiety or depression in the last month), a visual acuity (including corrected vision) of >0.8, and had no color blindness or color weakness. The participants were asked not to drink coffee or other drinks containing stimulants during the 24 h before the study. The experimental protocol was approved by the university ethics committee (approval number: HR 125-2019), and all subjects provided written informed consent.

### 2.2. Stimuli

#### 2.2.1. Imitation Task

The imitation task used in this study involved single gesture imitation. According to Makuuchi and Pizzamiglio et al. [16,17], we prepared 70 different hand gestures, including those involving the left hand, right hand, and both hands. We used finger coordination and palm orientation as variables, and generated 70 gesture images (25 for the left hand, 25 for the right hand, and 20 for both hands). We conducted a pilot study in which we asked four subjects to imitate the gestures and evaluate the pictures, and as a result, 10 difficult gesture pictures were excluded. The remaining 60 gesture pictures (including 18 for the left hand, 23 for the right hand, and 9 for both hands) were used in the imitation task.

**Figure 1:**
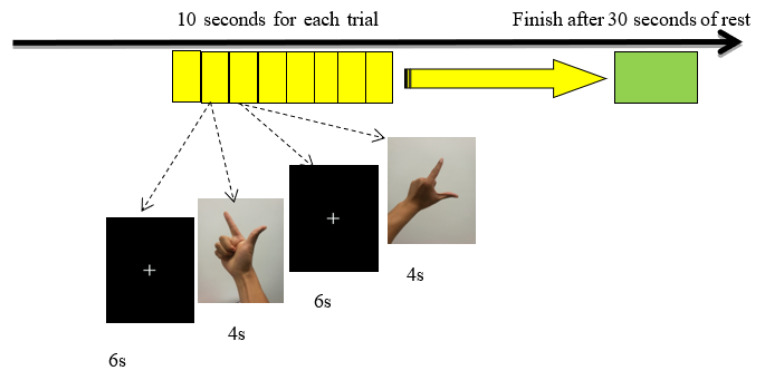
Flow chart of the imitation tasks.

We used the E-Prime software to administer the action simulation task. The task consisted of 60 10-s trials presented on a screen in a pseudo-random order. Each trial included a fixation point (6S) and a gesture imitation task map (4S). When a new gesture imitation task appeared, the subjects were required to imitate the movement as fast as possible and to then press the ‘confirm’ key on a keyboard. After all of the 60 tasks were completed, the screen indicated that the participants could rest for 30 s, and the task ended (as shown in Figure 1). The hand gesture imitation task was divided into three experimental conditions: left hand imitation, right hand imitation, and double hand imitation. In order to facilitate the supervision of the participants, as well as the evaluation of task performance, video recording was conducted during the experiment.

In previous studies on imitation, the speed of imitation [18], accuracy [19], error (spatial error, body part error, etc.) [19,20] have been used to evaluate imitation, along with auxiliary tools (such as motion capture instruments) [21]. Due to the fact that previous studies have indicated that accuracy and imitation speed are effective measures of imitation, we evaluated these variables in the present study.

#### 2.2.2. Motor Coordination Test—Harre Circuit Test (HCT)

**Figure 2:**
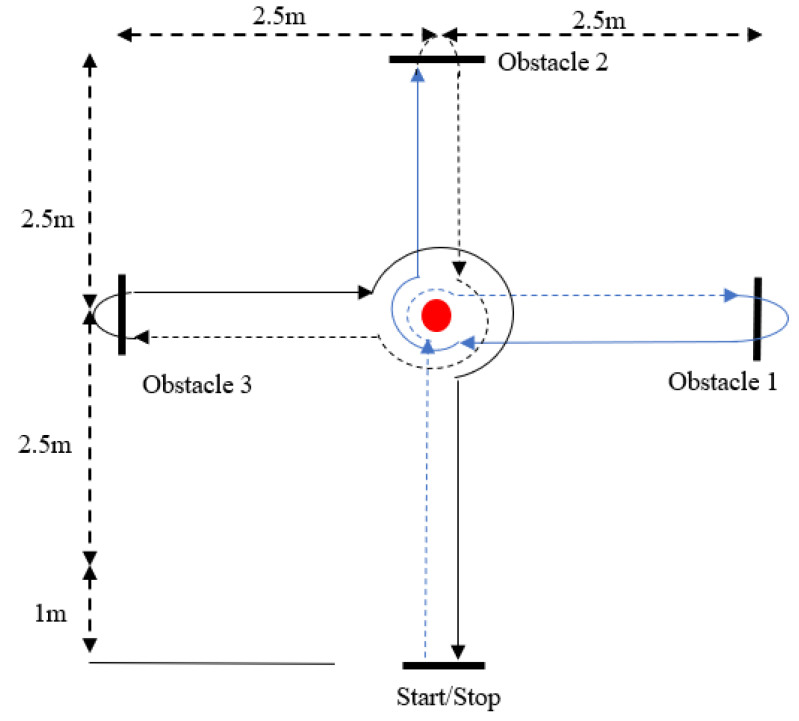
Harre circuit test (HCT).

The HCT is a measure of dynamic motor coordination, coordinative ability, and cognitive capabilities, which is general and effective in measuring coordination ability. Specifically, subjects are required to move through a circuit (Figure 2) as quickly as possible. After completing a 10-min warm-up activity, the subjects were asked to start running 0.7 m from a starting point, and to move through the circuit as directed. Timing began when the participants arrived at the starting point. They were instructed to enter the circuit at the fastest speed possible, and to move through the circuit in a particular pattern to reach the end point. If the subjects made mistakes (stepping on the center point, crossing the obstacles in the wrong order, etc.) or touched any of the obstacles during the test (e.g., marker posts), they were considered to have failed the test, and were asked to repeat the test after an interval of 5 min. Two valid scores were recorded (the interval between the two tests was 5 min) for each subject, and the shortest score was selected for statistical analysis [22,23].

### 2.3. Procedure

After arriving at the laboratory, the subjects completed a form to collect their demographic information and signed an informed consent form. After describing the detailed experimental process and related task requirements, the experiment began.

**Figure 3:**
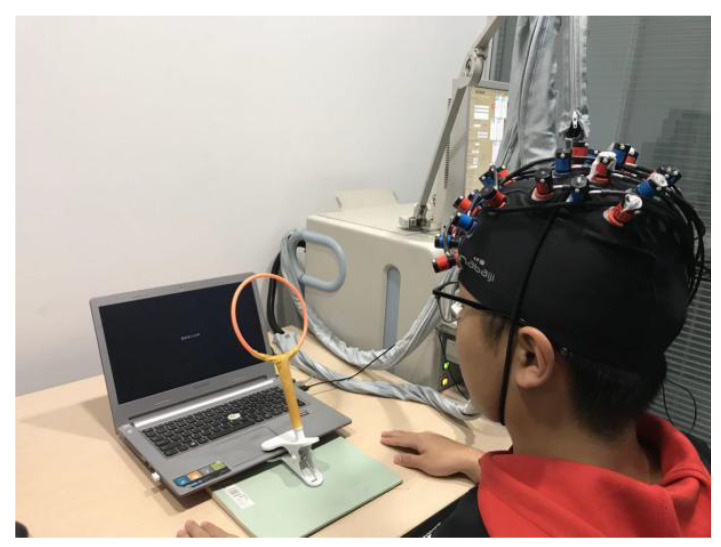
Experimental settings.

The subjects sat at a table in front of a computer, and were fitted with an *f*NIRS cap. After adjusting the channel signals in order to ensure a strong connection, the action simulation task began. When the subjects performed the imitation tasks, they were required to confine their actions to a designated area, which was indicated by a yellow circle positioned in front of the computer on the experimental table (as shown in Figure 3). Requiring the subjects to complete the imitation task in the designated place minimized the difference in the imitation time caused by different imitation positions. When the subjects completed the imitation task, they conducted the HCT in a specified test site.

### 2.4. f*NIRS* Data Acquisition

**Figure 4:**
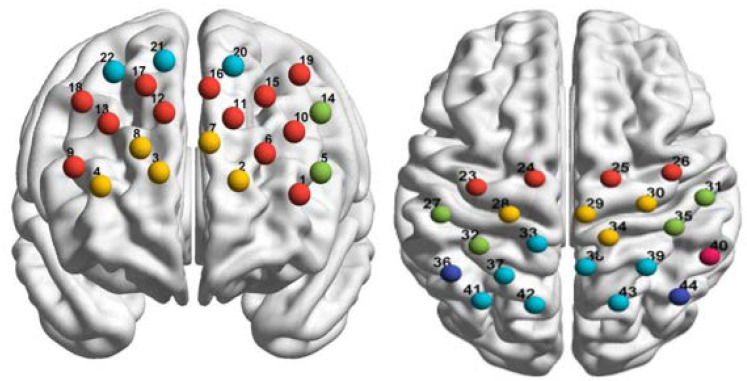
Schematic diagram of 44 channels over the frontal lobe and parietal lobe (**left**: frontal lobe; **right**: parietal lobe).

While the subjects were performing the imitation task, we used *f*NIRS (ETG-4000, Hitachi Medical Coopteration, Tokyo, Japan) to measure changes in signals representing oxyhemoglobin and deoxyhemoglobin concentrations in the brain. The sampling rate was 10 Hz. We used two 3 × 5 light pole probe patches, each with 8 emitters and 7 receivers. The probe spacing was 3 cm, and there were 44 channels in total, covering the frontal and parietal lobes, as shown in Figure 4. According to the international standard 10–20 system, the probe at the middle point of the lowest line of the plate was placed over FPZ, and the paths were placed horizontally. The specific spatial position of each channel was determined using a 3D locator.

Using the probability registration method, the *f*NIRS channel positions were reg-istered with the Montreal Neurological Institute spatial coordinates in order to obtain the corresponding locations of the Brodmann areas in Talairach coordinates. In this study, we examined activity in the dorsolateral prefrontal cortex (DLPFC, Left-DLPFC: ch1, ch 6, ch 10, ch 11, ch 15, ch 16, ch 19; Right-DLPFC: ch 9, ch 12, ch 13, ch 17, ch 18), frontopolar area (Left: ch 2, ch 7; Right: ch3, ch 4, ch 8), pars triangularis Broca’s area (PTR, Left-PTR: ch 5, ch 14), prefrontal eye movement area (Left: ch 20; Right: ch 21, ch 22), supplementary motor cortex (SMA, Left-SMA: ch 23, ch 24; Right-SMA: ch 25, ch 26), primary motor cortex (PMC, Left-PMC: ch 28; Right-PMC: ch 29, ch 30, ch 34), primary somatosensory cortex (S1, Left-S1: ch 27, ch 32; Right-S1: ch 31, ch 35), soma-tosensory association cortex (SSAC, Left-SSAC: ch 33, ch 37, ch 41, ch 42; Right-SSAC: ch 38, ch 39,ch 43), angular gyrus (ANG, Left-ANG: ch 36; Right-ANG: ch 44), and su-pramarginal gyrus (SMG, Left-SMG: ch 40), as shown in Figure 4.

As per previous studies on the neural mechanisms of imitation, we only analyzed activity in the MNS, including the SMA, PMC, S1, SSAC, ANG, and SMG.

### 2.5. Data Processing and Analysis

#### 2.5.1. Behavioral Data Processing

We used the E-Prime software to evaluate performance in the imitation tasks. We calculated the accuracy of gesture imitation and the average time for each trial under the right hand, left hand, and double hand imitation. A trial was scored as ‘correct’ if the gesture was completed accurately within 4 s of the trial onset. Otherwise, the trial was scored as ‘incorrect’. We calculated the reaction time as the average time taken for the subjects to press the ‘confirm’ key in ‘correct’ trials.

#### 2.5.2. *f*NIRS Data Processing and Analysis

We used the NIRS_SPM toolbox in MATLAB (Math-Works Inc., Natick, MA, USA) to process changes in blood-oxygen-dependent signals in the *f*NIRS data [24]. NIRS_SPM mainly uses the general linear model (GLM) to analyze *f*NIRS data. Data preprocessing included wavelet analysis and low-pass filtering in order to remove drift and noise, thus eliminating the influence of movements and physiological artifacts (such as heartbeats) on the *f*NIRS data. Second, according to the GLM model, we set the reference wave of oxygenated hemoglobin (HbO), deoxygenated hemoglobin (HbR) and total hemoglobin (HbT) changes in each channel. The time-dependent hemodynamic response function included three conditions: left hand imitation, right hand imitation, and double hand imitation. Finally, the model estimated the changes in HbO, HbR, and HbT concentration in the 44 channels, expressed as beta values. In order to obtain the neural activity in the cerebral cortex associated with imitation, we only analyzed brain activation in successful trials.

#### 2.5.3. Statistical Analysis

SPSS 26.0 (SPSS Inc., Chicago, IL, USA) was used to analyze the relationship between imitation and motor coordination. Independent sample *t* tests were used to compare motor coordination between the male and female subjects. Pearson correlation analysis was used to calculate the correlation between the data of imitation behavior and the activation and motor coordination of imitation-related brain areas.

## 3. Results

We recruited a total of 53 subjects (24 men and 29 women). As obesity will affect the performance of the coordination ability, the results will cause great interference, so the obese subjects were deleted. According to the World Health Organization definition of obesity [25] (BMI values ≥ 24.0), seven subjects (six men and one woman) with BMI values ≥ 24.0 were excluded from the analysis. Finally, data from 46 subjects were analyzed. The demographic information is given in Table 1.

### 3.1. Behavioral Results

The descriptive statistics regarding motor coordination and imitation ability are given in Table 2 below.

The results showed a significantly positive correlation between the average time taken to complete the imitation tasks in the right hand (*r* = 0.30, *p* = 0.04), left hand (*r* = 0.32, *p* = 0.02), and double hand trials (*r* = 0.39, *p* = 0.01). A correlation test revealed a significant correlation between motor coordination and imitation ability, such that higher motor coordination was associated with a shorter imitation time.

On this basis, we used gender as a grouping variable, and conducted an independent sample *t* test to identify differences in motor coordination and imitation between male and female participants. The results showed significant differences in motor coordination between men and women (*t*_(44)_ = −3.18, *p* = 0.003, Cohen’s *d* = −0.97) such that motor coordination in men was significantly better than that in women.

**Figure 5:**
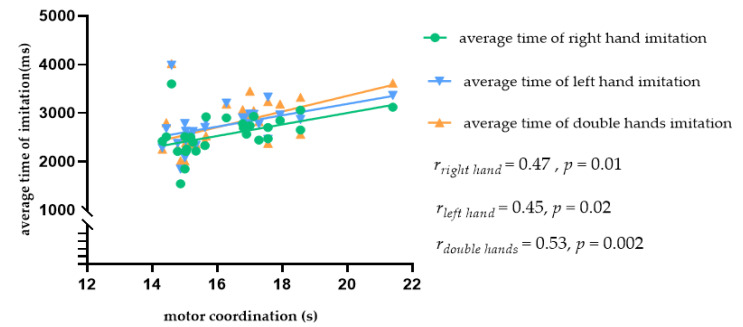
Scatter plot showing the relationship between motor coordination and the average imitation time in female students.

Given the significant gender differences in motor coordination, we analyzed the data from male and female participants separately. The results showed that, in female participants, there was a significantly positive correlation between motor coordination and the average time in the right hand imitation trials (*r*_right hand_ = 0.47, *p* = 0.01), left hand imitation trials (*r*_left hand_ = 0.45, *p* = 0.02), and double hand imitation trials (*r*_double hands_ = 0.53, *p* = 0.004). Thus, better motor coordination was linked with shorter imitation time in female students, as shown in Figure 5. Since we found no correlation between motor coordination and imitation in the male students, we did not further examine the data from this group.

### 3.2. Correlation Test between Imitation and Activation of Region of Interest (ROI)

**Figure 6:**
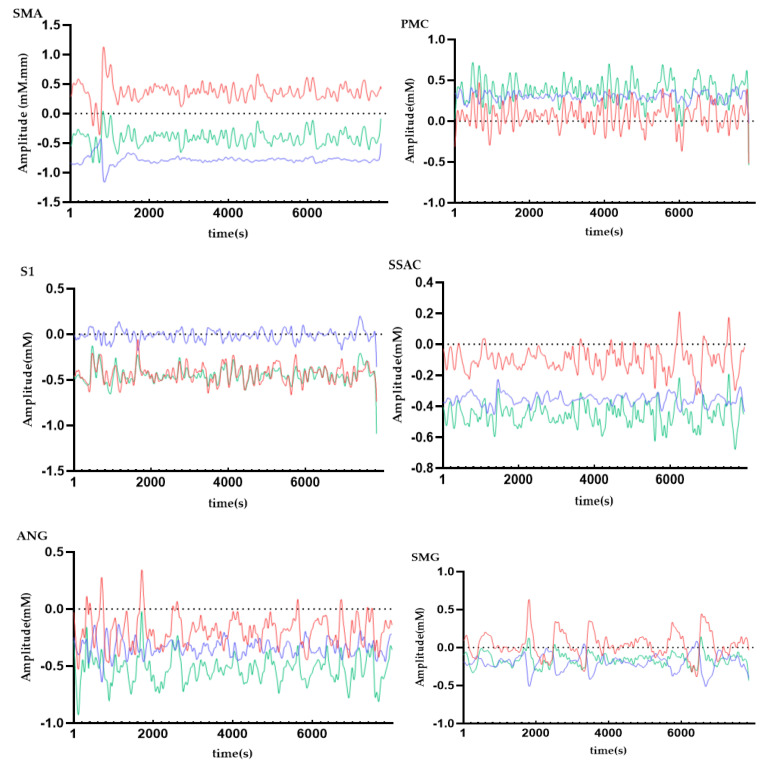
Time series of hemodynamic response for HbO (red line), HbR (blue line), and HbT (green line) across all subjects FAbbreviations: SMA = supplementary motor cortex; PMC = primary motor cortex; S1 = primary somatosensory cortex; SSAC = somatosensory association cortex; ANG = angular gyrus; SMG = supramarginal gyrus.

In order to observe the neural response to the task intuitively, we presented time series across all subjects from ROIs (Figure 6). In this figure, we can obviously see the task period yielded an increased hemodynamic response, as the amplitude of the HbO signal is higher than that of the HbR and HbT signal, so next we only analyzed HbO.

Due to the fact that we found no significant correlation between the accuracy of imitation and motor coordination in the behavioral results, we did not further analyze the imitation accuracy data. The correlation results are shown in Table 3. The average imitation time in women was negatively correlated with the activation degree in most regions of interest (ROIs), such that higher activation in the relevant ROI was associated with a stronger imitation ability.

### 3.3. The Moderating Effect of Motor Coordination

**Figure 7:**
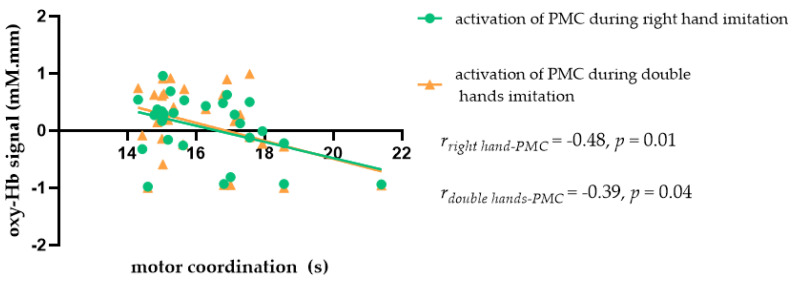
Scatter plot showing the relationship between motor coordination and MNS activation during imitation in female students.

In order to understand whether motor coordination modulated the relationship between imitation and ROI activation in the MNS, we first examined the correlation between motor coordination and ROIs in the MNS in the male and female participants. The results showed that motor coordination in women was significantly negatively correlated with PMC activation during right hand imitation (*r*_right hand-PMC_ = −0.48, *p* = 0.01) and double hand imitation trials (*r*_double hands-PMC_ = −0.39, *p* = 0.04), as shown in Figure 7. Given the significant correlation between motor coordination, imitation, and MNS activity, as well as the data indicating that different types of motor coordination may have different effects on imitation, we speculated regarding the ways in which motor coordination might influence the relationship between imitation and MNS activation.

We regarded MNS activation as an independent variable, motor coordination as a moderating variable, and imitation as a dependent variable. Our data indicate that motor coordination influenced the relationship between ROI activation and imitation in female participants only. See Table 4 for details regarding the moderating effect and simple slope analysis results.

From the above table, one can see that when low motor coordination (*M* = 17.53) was associated with higher MNS activation (PMC and S1 for right hand, left hand, and double hand trials and SMA, ANG, and SMG for left hand trials), female students exhibited a better imitation ability. Further, when motor coordination was high (*M* = 14.96), MNS activation did not significantly affect imitation.

## 4. Discussion

In this study, we explored the relationship between motor coordination and imitation, as well as the moderating role of motor coordination in terms of behavior and surface neural activation. Our results revealed a significant positive correlation between motor coordination and behavioral performance in the right hand, left hand, and double hand trials in female students. Further analysis indicated that motor coordination in female students moderated the relationship between MNS activation and imitation behavior. For female students with low motor coordination, ROI activation was significantly correlated with imitation performance. Thus, higher MNS activation was associated with greater action imitation in this population. However, we found no relationship between ROI activation and action imitation in women with higher motor coordination.

We found a positive correlation between motor coordination and the average imitation time in female students. Thus, women with better motor coordination had a stronger action imitation ability. This is consistent with the results of Kamandulis et al. [26], who examined basketball skills in 17-year-old participants, and found that basketball players with better motor coordination acquired new basketball skills faster. This phenomenon may be related to a strong imitation ability, because motor coordination can facilitate the integration between different systems, body parts, and organs during imitation. Thus, individuals with good motor coordination are likely to have a strong imitation ability. In the present study, this result was only observed in female subjects, and not in male subjects. We speculate that this may be due to the increased likelihood of boys participating in sports activities, which can improve motor coordination during development.

We also found that the PMC, ANG, SMG, and other MNS ROIs were significantly activated during imitation. Previous studies have indicated that the MNS is activated during imitation, mainly in the PMC, ANG, SMG, and SSAC [11,27]. In our participants, the average imitation time was negatively correlated with the degree of MNS activation, which indicated that the subjects achieved a better imitation performance if they had stronger internal processing. Thus, our study verified that brain regions associated with the MNS were activated during imitation.

This study found that higher motor coordination in healthy female college students was associated with higher activation in the PMC and a stronger action imitation ability. Kiyama et al. also found that motor coordination was related to the activation of the PMC during imitation [28], and that motor coordination was closely related to the function of the PMC. The PMC is an important brain area for action control and execution [29]. As the core brain area of the MNS, it is the neural basis of action imitation [30]. Our behavioral and neuroimaging findings supported the close relationship between motor coordination, action imitation, and PMC activity in healthy female subjects. As a next step, future studies should test the regulatory role of motor coordination on the interaction between ROI activation and the action imitation.

In this study, we found that motor coordination in female students moderated the interaction between brain activation and imitation ability. Specifically, in female students with lower motor coordination, higher activation in regions involved in the MNS, such as the PMC, SMA, S1, ANG, and SMG, when they imitated actions with their right hand, left hand, and both hands, was associated with better action imitation. However, this relationship was not found in women with higher motor coordination. We speculate that the women with higher motor coordination might have participated in more sports, thus enhancing their action imitation ability. During imitation, the efficiency of neural resources related to the MNS was high, and general nervous system activation was low. Thus, it appears that the degree of MNS activation cannot be used to predict the action imitation ability. However, women with low motor coordination may need to recruit more MNS-related resources in order to efficiently complete an imitation task. Therefore, in this population, higher MNS activation may be related to better imitation.

Several studies have pointed out that many regions in the MNS are activated during imitation; specifically, the SMG, PMC, SMA, S1, SSAC, and ANG [11,27]. Our study confirmed this with respect to the PMC, ANG, SMG, and other MNS ROIs. Thus, this study once again verified that brain regions associated with the MNS are activated during imitation via the use of *f*NIRS. When obtaining *f*NIRS data, the changes in HbO and HbR concentrations were measured simultaneously. However, there is controversy about which signal to choose in order to analyze the brain hemodynamic response. In this study, we mainly focus on the HbO signal, because it is usually observed that its amplitude is higher than that of the HbR and HbT signal [31,32]. In other words, the signal-to-noise ratio of HbO is better, and the signal is more sensitive to the task response [33]. Similar to the results of previous studies, it was found that HbO is a more effective measurement [30,34].

There are also some other important findings in this study. In this study, there is no correlation between the imitation and motor coordination of men. This study speculates that the possible reason is that men participate in more sports activities, and the coordination ability is more affected by heredity or congenital. This view is only the assertion of this study. In the future, we will further study the relationship between action imitation and coordination ability.

## 5. Conclusions

We found a significantly positive correlation between motor coordination and the average imitation time, such that when motor coordination was low, a higher degree of activation in the MNS was associated with a better imitation ability. However, when the motor coordination is high, the degree of MNS activation has no relation to imitation. This study reveals the relationship between action imitation and coordination ability from two aspects of behavior and brain hemodynamics, and supplements the related research on the relationship between the two in the adult population.

## Figures and Tables

**Table 1:** Demographic characteristics of subjects (*M* ± *SD*).

Variables	Male (N = 18)	Female (N = 28)	Total (N = 46)
Age (years)	19.44 ± 0.51	19.64 ± 0.95	19.57 ± 0.81
Height (cm)	178.17 ± 6.95	161.36 ± 4.50	167.93 ± 9.96
Weight (kg)	72.56 ± 11.69	54.71 ± 5.82	61.70 ± 12.22
BMI (kg/m^2^)	21.77 ± 2.72	20.99 ± 1.80	21.69 ± 2.35

**Table 2:** Descriptive statistics of motor coordination and imitation (*M* ± *SD*).

Behavioral Indicators	Male (N = 18)	Female (N = 28)	Total (N = 46)
Motor coordination(s)	14.74 ± 1.47	16.25 ± 1.63	16.66 ± 1.72
Imitation ability			
Accuracy in right hand trials (%)	98.47 ± 2.12	99.26 ± 1.96	98.95 ± 2.04
Average time in right hand trials (ms)	2583.798 ± 504.22	2558.56 ± 407.93	2568.43 ± 442.77
Accuracy in left hand trials (%)	97.72 ± 2.81	97.89 ± 4.01	97.82 ± 3.55
Average time in left hand trials (ms)	2745.00 ± 511.56	2750.41 ± 423.64	2748.29 ± 454.48
Accuracy in double hand trials (%)	96.30 ± 5.39	97.62 ± 5.54	97.10 ± 5.46
Average time in double hand trials (ms)	2745.23 ± 537.31	1749.60 ± 497.50	2747.89 ± 507.52

**Table 3:** Correlation between average imitation time and MNS activation in female students.

ROI	Right Hand Imitation	Left Hand Imitation	Double Hand Imitation
SMA	−0.37 *	−0.42 *	−0.52 **
PMC	−0.46 *	−0.27	−0.41 *
S1	−0.40 *	−0.50 **	−0.53 **
SSAC	−0.35	−0.31	−0.48 *
ANG	−0.43 *	−0.45 *	−0.46 *
SMG	−0.36	−0.47 *	−0.56 **

* denotes a *p*-value less than 0.05; ** denotes a *p*-value less than 0.01. Abbreviations: SMA = supplementary motor cortex; PMC = primary motor cortex; S1 = primary somatosensory cortex; SSAC = somatosensory association cortex; ANG = angular gyrus; SMG = supramarginal gyrus.

**Table 4:** Motor coordination moderates the relationship between MNS activity and imitation behavior in female students.

Independent Variable		Effect	*t*	95%CI	*p*
PMC activation during right hand imitation trials	Mean − 1SD	−463.89	−2.71	[−816.94, −110.84]	0.01
	Mean	−200.05	−1.52	[−472.20, 72.10]	0.14
	Mean + 1SD	63.79	0.34	[−317.46, 445.04]	0.73
S1 activation during right hand imitation trials	Mean − 1SD	−459.20	−2.80	[−797.91, −120.49]	0.01
	Mean	−249.84	−2.14	[−490.42, −9.27]	0.04
	Mean + 1SD	−40.49	−0.29	[−327.59, 246.62]	0.77
SMA activation during left hand imitation trials	Mean − 1SD	−1191.12	−3.22	[−1953.96, −428.27]	0.004
	Mean	−802.73	−3.11	[−1335.86, −269.60]	0.005
	Mean + 1SD	−414.34	−1.96	[−850.35, 21.67]	0.06
S1 activation during left hand imitation trials	Mean − 1SD	−617.88	−3.62	[−969.98, −265.77]	0.001
	Mean	−373.91	−3.16	[−618.19, −129.63]	0.004
	Mean + 1SD	−129.94	−0.97	[−406.15, 146.26]	0.34
ANG activation during left hand imitation trials	Mean − 1SD	−1059.95	−5.82	[−1435.94, −683.96]	0.001
	Mean	−421.16	−2.85	[−725.73, −116.59]	0.01
	Mean + 1SD	217.63	0.82	[−327.96, 763.22]	0.42
PMC activation during left hand imitation trials	Mean − 1SD	−535.90	−3.55	[−847.32, −224.47]	0.002
	Mean	−255.04	−2.06	[−510.67, 0.59]	0.05
	Mean + 1SD	25.81	0.14	[−368.85, 420.47]	0.89
SMG activation during left hand imitation trials	Mean − 1SD	−1150.46	−4.83	[−1641.47, −659.25]	0.001
	Mean	−716.24	−4.41	[−1051.45, −381.03]	0.002
	Mean + 1SD	−282.12	−1.58	[−651.15, 86.90]	0.13
PMC activation during double hand imitation trials	Mean − 1SD	−469.85	−2.71	[−827.96, −111.74]	0.01
	Mean	−191.51	−1.55	[−447.26, −64.24]	0.13
	Mean + 1SD	86.82	0.52	[−259.51, 433.16]	0.52
S1 activation during double hand imitation trials	Mean − 1SD	−469.85	−2.71	[−827.96, −111.74]	0.01
	Mean	−191.51	−1.55	[−447.26, 64.24]	0.13
	Mean + 1SD	86.82	0.52	[−259.51, 433.16]	0.61

Abbreviations: SMA = supplementary motor cortex; PMC = primary motor cortex; S1 = primary somatosensory cortex; SSAC = somatosensory association cortex; ANG = angular gyrus; SMG = supramarginal gyrus.

## Data Availability

The data presented in this study are available on request from the corresponding author.
